# Arctic and Antarctic forcing of ocean interior warming during the last deglaciation

**DOI:** 10.1038/s41598-023-49435-0

**Published:** 2023-12-16

**Authors:** Joseph A. Stewart, Laura F. Robinson, James W. B. Rae, Andrea Burke, Tianyu Chen, Tao Li, Maria Luiza de Carvalho Ferreira, Daniel J. Fornari

**Affiliations:** 1 School of Earth Sciences University of Bristol, Queens Road, Bristol, BS8 1RJ UK; 2 Department of Environment and Geography, University of York, York, UK; 3 School of Earth and Environmental Sciences, University of St Andrews, St Andrews, KY16 9TS UK; 4 School of Earth Sciences and Engineering, Nanjing University, Nanjing, 210023 China; 5 Key Laboratory of Palaeobiology and Petroleum Stratigraphy, Nanjing Institute of Geology and Palaeontology, Chinese Academy of Sciences, Nanjing, China; 6 Woods Hole Oceanographic Institution, Falmouth, MA USA

**Keywords:** Ocean sciences, Palaeoceanography, Palaeoclimate

## Abstract

Subsurface water masses formed at high latitudes impact the latitudinal distribution of heat in the ocean. Yet uncertainty surrounding the timing of low-latitude warming during the last deglaciation (18–10 ka) means that controls on sub-surface temperature rise remain unclear. Here we present seawater temperature records on a precise common age-scale from East Equatorial Pacific (EEP), Equatorial Atlantic, and Southern Ocean intermediate waters using new Li/Mg records from cold water corals. We find coeval warming in the tropical EEP and Atlantic during Heinrich Stadial 1 (+ 6 °C) that closely resemble warming recorded in Antarctic ice cores, with more modest warming of the Southern Ocean (+ 3 °C). The magnitude and depth of low-latitude ocean warming implies that downward accumulation of heat following Atlantic Meridional Overturning Circulation (AMOC) slowdown played a key role in heating the ocean interior, with heat advection from southern-sourced intermediate waters playing an additional role.

## Introduction

**Figure 1:**
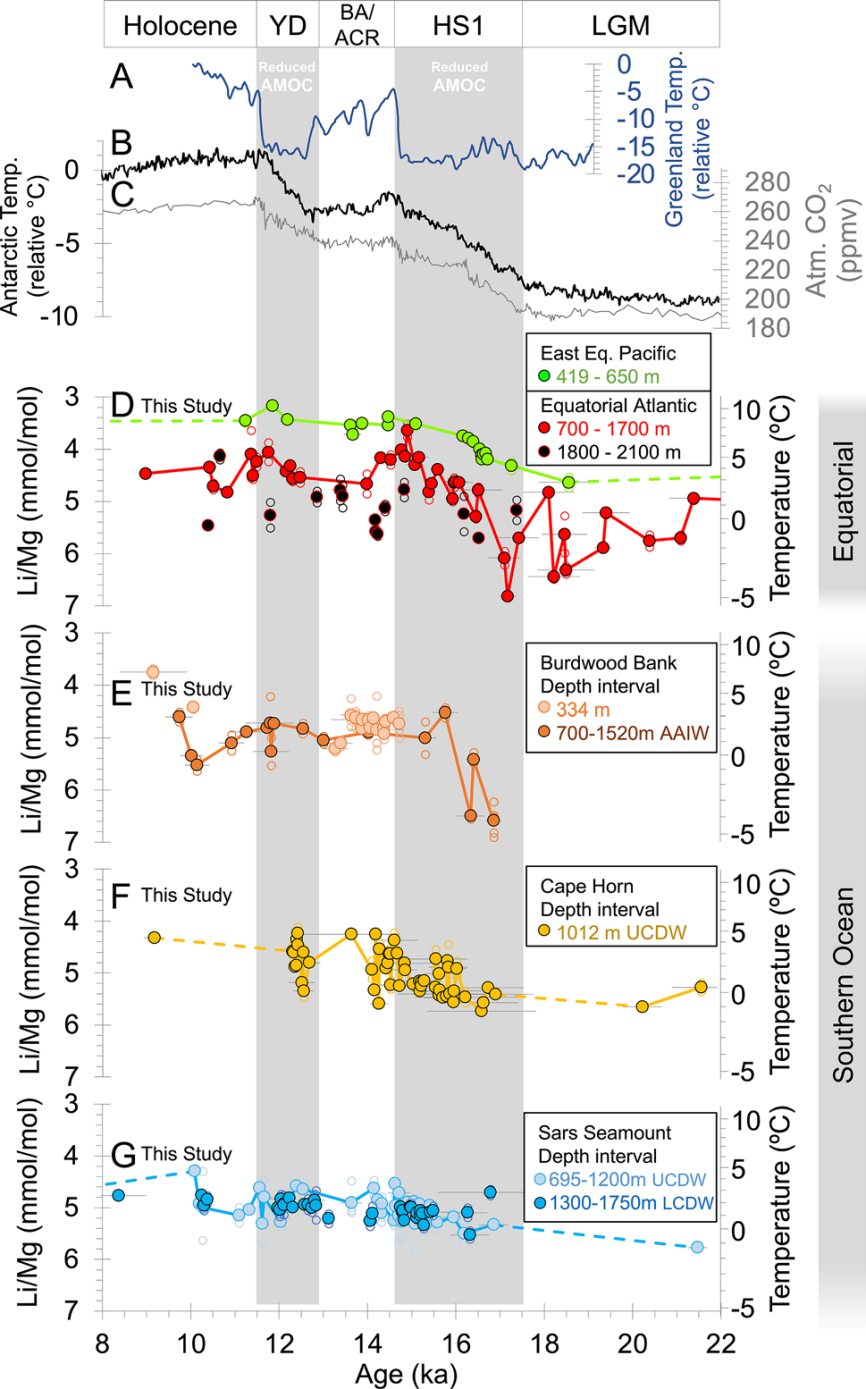
Coral Li/Mg results compared to ice core data. (**A**) Greenland and (**B**) Antarctic ice core stable isotope records of deglacial temperature change (stacked stable isotopes^1,2,63^). (**C**) Antarctic ice core atmospheric CO_2_ record^64^. New deglacial cold-water Li/Mg temperature records from (**D**) East Equatorial Pacific and Equatorial Atlantic, (**E**) Burdwood Bank (AAIW), (**F**) Cape Horn (UCDW), and (**G**) Sars/Interim sites (UCDW and LCDW). The Younger Dryas and Henrich Stadial 1 are highlighted by grey bars. Replicate measurements (open symbols) agree well within coral specimens; the majority yielding seawater temperature values within 2 °C of each other. Replicate averages shown as filled symbols. Error bars denote 2σ age uncertainties. Analytical error is smaller than the markers shown.

**Figure 2:**
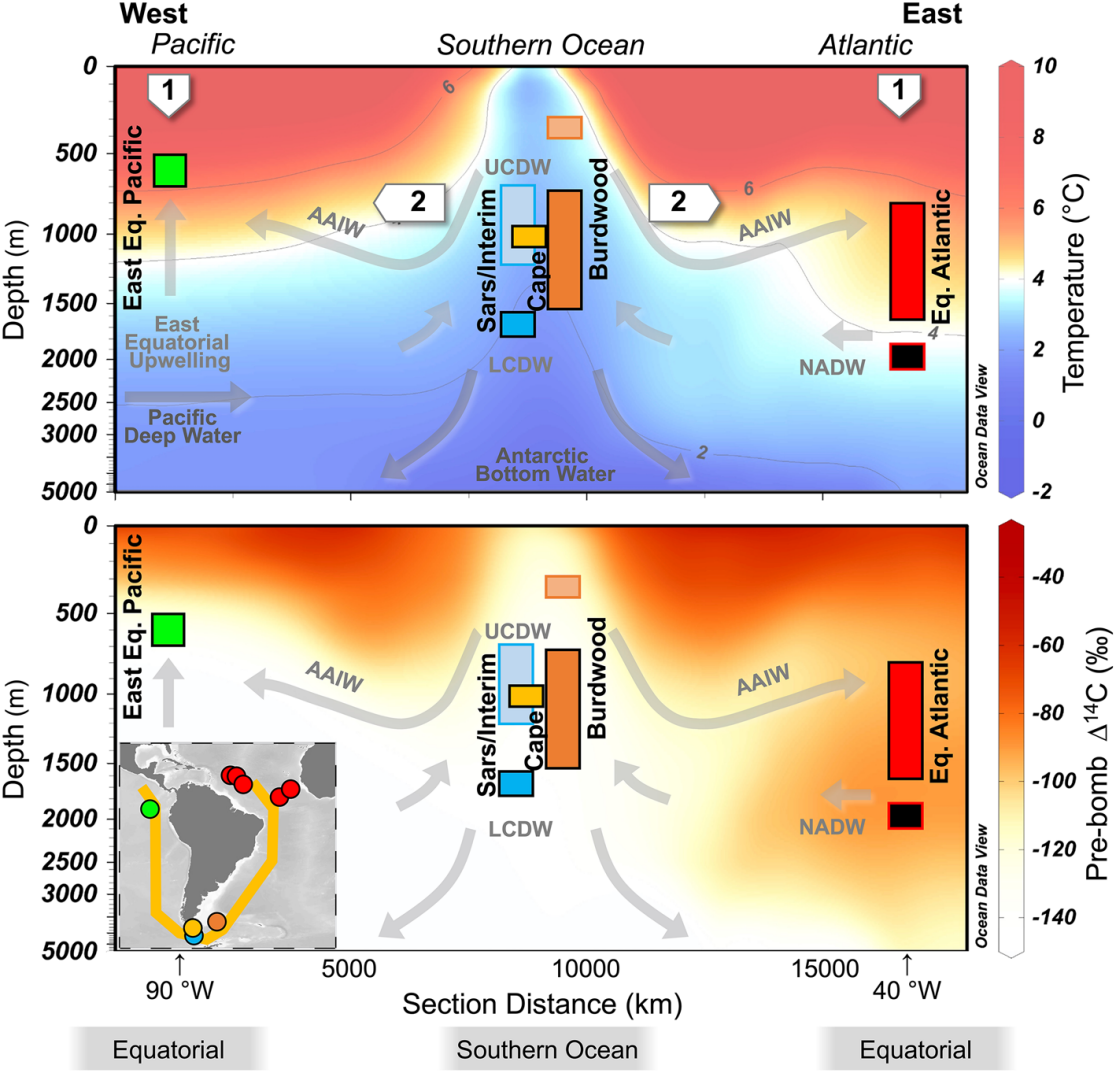
Cold-water coral sample locations in the Equatorial Atlantic (Cruise JC094; red), East Equatorial Pacific (Cruises MV1007 and NA064; green) and Drake Passage (Cruises NBP0805 and NBP1103; Burdwood Bank, orange; Cape Horn, yellow; and Sars Seamount, blue). Depth section (yellow line in insert map) showing grouping of coral samples in this study by water depth. Modern temperature and pre-bomb radiocarbon content of seawater highlight the strong temperature and ^14^C depth gradient in the Pacific and the northward flowing, radiocarbon-depleted AAIW, in the Atlantic^53^. Temperature contours for 2, 4 and 6 °C are shown as black lines. Numbered arrows refer to warming mechanisms referred to in the text: Warming 1, Downward mixing of heat; Warming 2, Advected heat via southern-sourced intermediate waters. Plots created using Ocean Data View^65^.

Precisely-dated ice core records from Greenland^1^ and Antarctica^2,3^ reveal that stepped high-latitude warming associated with atmospheric CO_2_ rise during the last deglaciation occurred in anti-phase between hemispheres (i.e. the “bipolar seesaw”^4^; Fig. 1). Northern Hemisphere cooler intervals, Heinrich Stadial 1 (HS1; ~ 16 ka) and the Younger Dryas (YD; ~ 12 ka), are linked to suppressed AMOC^5–7^ and disruption of latitudinal ocean heat exchange^8^, but are also associated with significant Antarctic warming (Fig. 1)^2^. The Southern Ocean is thought to have played a role in this warming and associated CO_2_ rise, providing a connection to the vast reservoirs of heat and carbon in the ocean’s interior via regional upwelling, and formation of deep and intermediate waters such as Antarctic Intermediate Water (AAIW)^9,10^. These southern-sourced intermediate waters are in turn thought to be important conduits for interhemispheric-transmission of these Antarctic climate signals (Fig. 2;^11,12^), though warming in the subsurface at these times may also occur due to decreased influence of cold waters from high northern latitudes^8,13^. Suggested mechanisms of deglacial low-latitude ocean warming therefore generally fall into two categories (Fig. 2): (1) a downward accumulation of heat from surface waters when cooling of the ocean interior by deep northern water masses is diminished during AMOC slowdown (e.g. reduction in cold North Atlantic Deep Water (NADW);^8,13–16^), and (2) intermediate water advection of heat from a warming Southern Ocean as a result of increased greenhouse gas concentrations (e.g. “ocean tunnelling” of southern-sourced intermediate waters;^11,12,16,17^).

**Figure 3:**
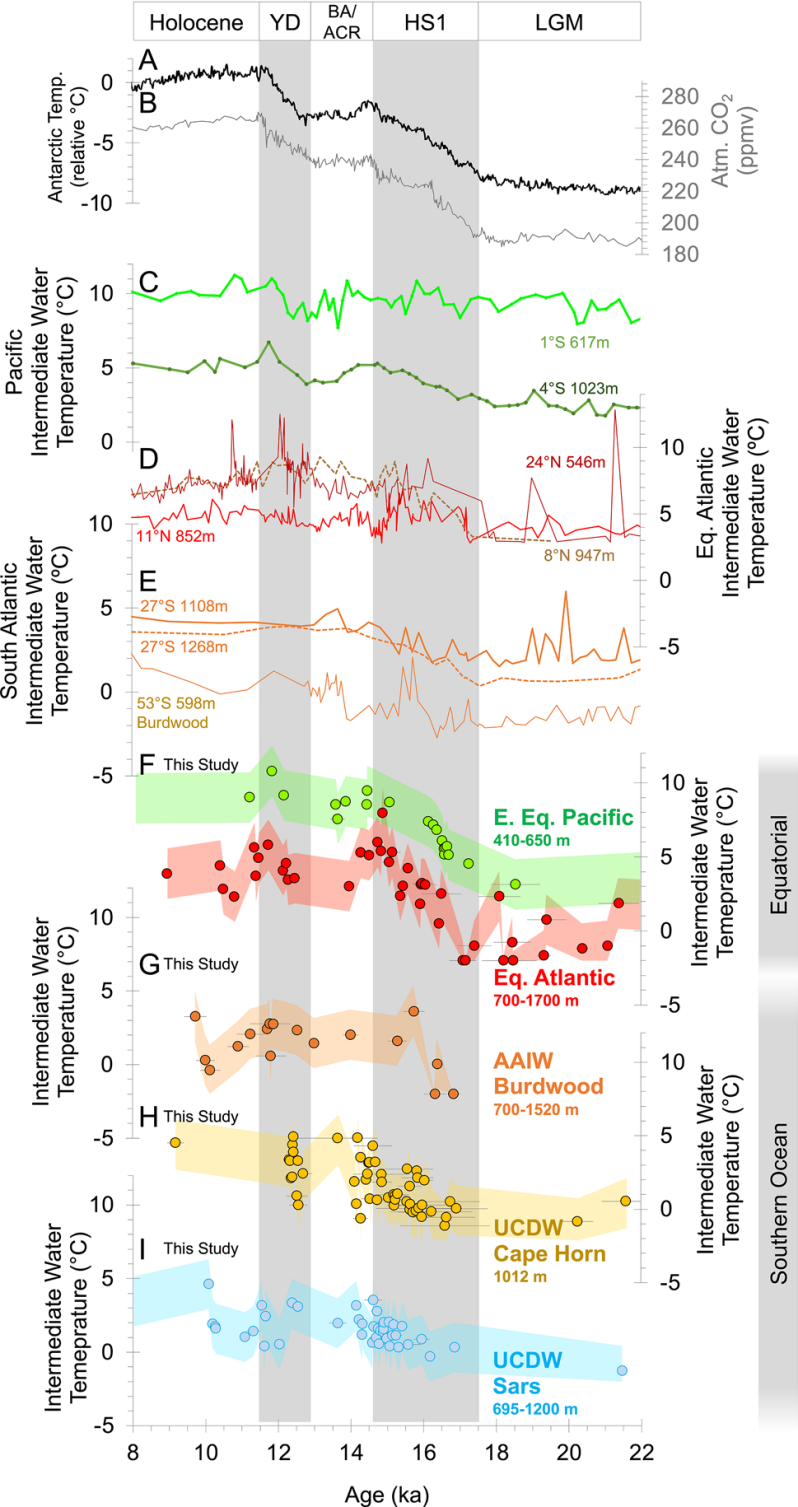
Coral temperature reconstruction compared to previous deglacial records. (**A**) Antarctic ice core temperature change and (**B**) atmospheric CO_2_ records^2,63,64^. (**C**) Previous East Equatorial Pacific intermediate water temperature records from benthic foraminifera δ^18^O^25^. (**D** and **E**) Examples of previous Equatorial and South Atlantic intermediate water temperature records from benthic foraminifera Mg/Ca and Li/Mg including: M78/1 235-1 (11° N, 852 m)^17^, KNR166-2-26JPC (24° N, 546 m)^23^, KNR197-3-46CDH (8° N, 947 m)^22^, KNR159‐5 ‐90GGC and -GGC36 (27° S, 1108 m and 1268 m)^16^, GC528 (53° S, 598 m)^19^. Coral Li/Mg intermediate water temperature reconstruction (this study) for (**F**) East Equatorial Pacific and Equatorial Atlantic, (**G**) Burdwood Bank (AAIW), (**H**) Cape Horn and (**I**) Sars/Interim (Upper Circumpolar Deep Water) sites. Error envelopes on temperature reconstructions denote the ± 1.7 °C (1σ) prediction interval uncertainty on the Li/Mg calibration by Stewart et al.^35^.

The relative importance of these two warming mechanisms is debated, therefore understanding the timing of events and the role of ocean circulation in the transmission of high-latitude climate anomalies continues to be an important research goal (Fig. 3^13,16–23^). However existing deglacial intermediate water records from the Atlantic do not agree on the timing and magnitude of temperature change. For instance, it is unclear whether warming commenced before^18,23^ or after^16,17,22^ the onset of HS1 at ~ 17 ka or the importance of warming during HS1^16,17^ or the YD^18,23^ (Fig. 3D).

There are also limited records of intermediate water temperature from the South Atlantic and Southern Ocean, therefore links between low-latitude ocean warming and ice core temperatures are difficult to establish (Fig. 3E). For example, benthic foraminifera temperature data from Burdwood Bank in the Drake Passage (Core GC528; 598 m water depth) suggest that warming happened abruptly, mid-way through HS1^19^, whereas records from further north on the Brazil Margin (1108 and 1268 m water depth) suggest that warming commenced at the start of HS1 and proceeded gradually during the deglaciation^16^.

Deglacial intermediate water temperature records from the EEP are also complex^24,25^. Temperature reconstruction from benthic foraminifera δ^18^O at 1023 m water depth suggest that + 3.5 °C deglacial warming of EEP intermediate waters likely occurred in step with Antarctic temperature change, whereas shallower records (617 m) do not indicate warming, suggesting little downward accumulation of heat (Fig. 3C;^25^).

The links between high-latitude climate and low-latitude Atlantic and Pacific intermediate waters are particularly hindered by uncertainty in deglacial circulation patterns. For instance, particularly radiogenic values recorded in seawater neodymium isotope reconstructions have been interpreted as northward incursion of southern-sourced intermediate waters into the Atlantic basin at 1330 m depth (12° N^26^) during HS1 and YD (Supplementary Fig. 1). However, other neodymium isotope records from shallower Atlantic and Caribbean sites (500–1000 m depth^27,28^) contradict this interpretation, suggesting that HS1 and YD were intervals of diminished southern-sourced intermediate waters. Efforts to reconcile these disparities through modelling propose that only the shallower core sites (< 1000 m depth) were influenced by an AAIW-like water mass during the last deglaciation, and southern-sourced intermediate water advection declined during times of weak AMOC^29^. Similarly, benthic foraminifera Cd/Ca nutrient proxy records from the Atlantic argue both for^30,31^ and against^32,33^ the presence of nutrient-rich intermediate waters during HS1 and YD (Supplementary Fig. 1). However, the influences of local inputs and remineralisation complicate the assessment of whether these nutrient peaks indeed indicate the presence of a southern-sourced water mass^34^. Therefore, in the absence of definitive circulation records, reliable temperature records on an accurate and precise age-scale, covering key high and low latitude sites, are needed to establish the timing, patterns and likely causes of deglacial sub-surface warming.

Sub-fossil cold-water scleractinian corals can be precisely radiometrically dated and contain a host of paleoceanographic proxy information. The Li/Mg ratio of their skeletal aragonite is strongly related to calcification temperature, while minimally impacted by species or calcification process^35^. We present a coherent dataset of coral Li/Mg-based temperature reconstructions from uranium–thorium dated corals^36–41^ collected from intermediate waters from the East Equatorial Pacific, Equatorial Atlantic and Southern Ocean (Drake Passage) over the last deglacial interval (Figs. 1, 2). We compare these temperature records with radiocarbon records measured previously in the same corals^36,38–40^ that provide a complementary tool to identify the presence of radiocarbon-depleted water masses at low-latitude sites. Collectively these coral records provide definitive constraints on the timing and magnitude of deglacial seawater temperature rise.

## Results and discussion

The most salient feature of our East Equatorial Pacific and Equatorial Atlantic coral temperature records is the close correspondence they bear to each other and to deglacial records of Antarctic temperature^2^, particularly during HS1 (Fig. 3; Supplementary Fig. 2). However, by exploring the details of these warming trends and comparing them to our Southern Ocean records we argue that, despite their similarity to the Antarctic, the magnitude and depth of warming implies that downward accumulation of heat (“Warming 1”; Fig. 2) was likely to have dominated the warming signal at these low-latitude sites.

### Low-latitude warming

The EEP corals (~ 600 m water depth) yield temperatures ~ 3 °C warmer than Equatorial Atlantic intermediate water samples (700–1700 m water depth), but both records commence warming at ~ 17 ka, with temperature increasing steadily throughout HS1 (~ + 6 °C increase). Our data offer lower temporal coverage after HS1, but potentially suggest a cooling/plateauing during the Bølling–Allerød (~ 14 ka), before increasing again (2–3 °C increase) during the YD interval (Figs. 1, 3). This pattern contrasts with Li/Mg results from deep samples (> 1800 m) in the Equatorial Atlantic that suggest temperatures varied by only 3.5 °C across the entire deglaciation (Fig. 1D).

**Figure 4:**
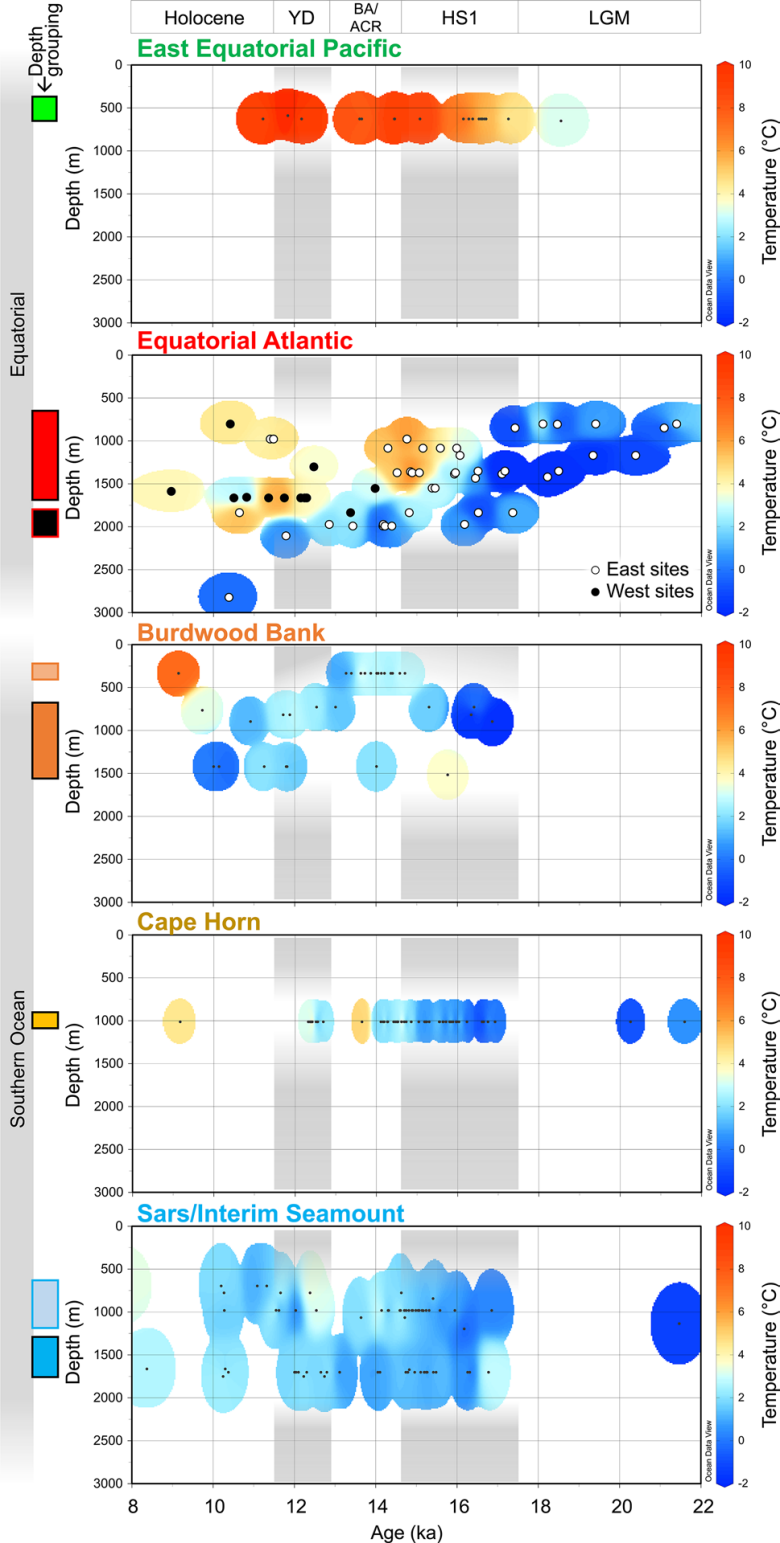
Hovmöller diagrams of coral temperature reconstruction with water depth. Li/Mg temperature proxy reconstruction for Equatorial and Southern Ocean sites in this study. Equatorial Atlantic sites are subdivided into East (Carter and Knipovich sites) and West sites (Vema Vayda and Gramberg sites). Plots created using Ocean Data View^65^.

To further explore the depth at which this deglacial warming occured, we display our reconstructed temperature data using Hovmöller diagrams (time versus depth; Fig. 4). The wide depth and time coverage of Equatorial Atlantic samples shows that the deglacial warming patterns discussed above occur as intense deepening of upper ocean warmth, particularly during late HS1 (15 ka) and late YD (11.5 ka). While strong warming (~ 5 °C) is observed at typical water depths of modern AAIW (~ 1000 m), this downward penetration of warm water also extends below ~ 1500 m, into water depths more typically associated with Glacial North Atlantic Intermediate Water or NADW^42^.

### Southern Ocean warming

Li/Mg ratios in our Southern Ocean corals also reveal sub-surface warming during the last deglaciation, however this occurs predominantly during the latter part of HS1 (16.5–14.5 ka; Figs. 1, 3). Coral records from Burdwood Bank below 700 m water depth are close to the source regions of AAIW and indicate that water temperatures were close to freezing (~ − 2 °C) in earliest HS1 (~ 17 ka), but warm abruptly by + 4 °C between 16.3 and 15.7 ka (Figs. 1E, 3G), synchronous with a rapid rise in atmospheric CO_2_ (Fig. 3B). This timing is consistent with previously observed changes in radiocarbon (Supplementary Fig. 1F^38^) and boron isotope (low pH;^41^) measurements in the same corals, and suggests that this warming was coincident with a pulse of convective mixing. These warmer temperatures at Burdwood Bank are largely maintained throughout the deglaciation, followed by cooling and warming in the earliest Holocene. This Burdwood Bank temperature record broadly agrees with previous estimates of intermediate water temperature based on nearby benthic foraminifera Mg/Ca recovered from comparable depths (598 m; Fig. 3E^19^). These foraminifera data suggest the bulk of a ~ 3 °C warming occurred during HS1 and, like our Burdwood Bank coral record, also show a notable warming centred on 16 ka (compare (Fig. 3E and G)). Where shallower dwelling corals (334 m water depth) are also available on Burdwood Bank (mainly during the Bølling–Allerød) reconstructed temperatures tend to be similar to deeper dwelling specimens (Fig. 1E).

Samples taken from Upper Circumpolar Deep Water (UCDW) from the Cape Horn area (1012 m; Figs. 1F, 3H) and the more southerly Sars Seamount sites (695 to 1200 m; Figs. 1G, 3I) offer better coverage during HS1 and show a more gradual and muted warming (+ 3 °C) than at Burdwood Bank. Deeper samples from the southerly Sars Seamount site that are currently at Lower Circumpolar Deep Water (LCDW) depths (> 1300 m; Fig. 1G) show very little deglacial warming (~ + 1 °C increase). This contrast is likely a result of more limited communication between LCDW and the surface, compared to the shallower and more northerly sites, which occupy less dense isopycnals in the Subantarctic Zone (27.4 vs 27.8 kg/m^3^).

### Mechanisms of warming and implications

Our data show distinctive warming of the Equatorial Atlantic and the EEP during HS1 (Fig. 3F), at a time of reduced AMOC, cold surface conditions in the North Atlantic, and warming of Antarctica alongside rising CO_2_ (Fig. 3A and B). We also show that these equatorial events occurred as the Southern Ocean warmed (particularly during HS1), albeit with a somewhat different structure and in a more muted fashion at the southern sites that occupy deeper isopynals (Fig. 3G–I).

**Figure 5:**
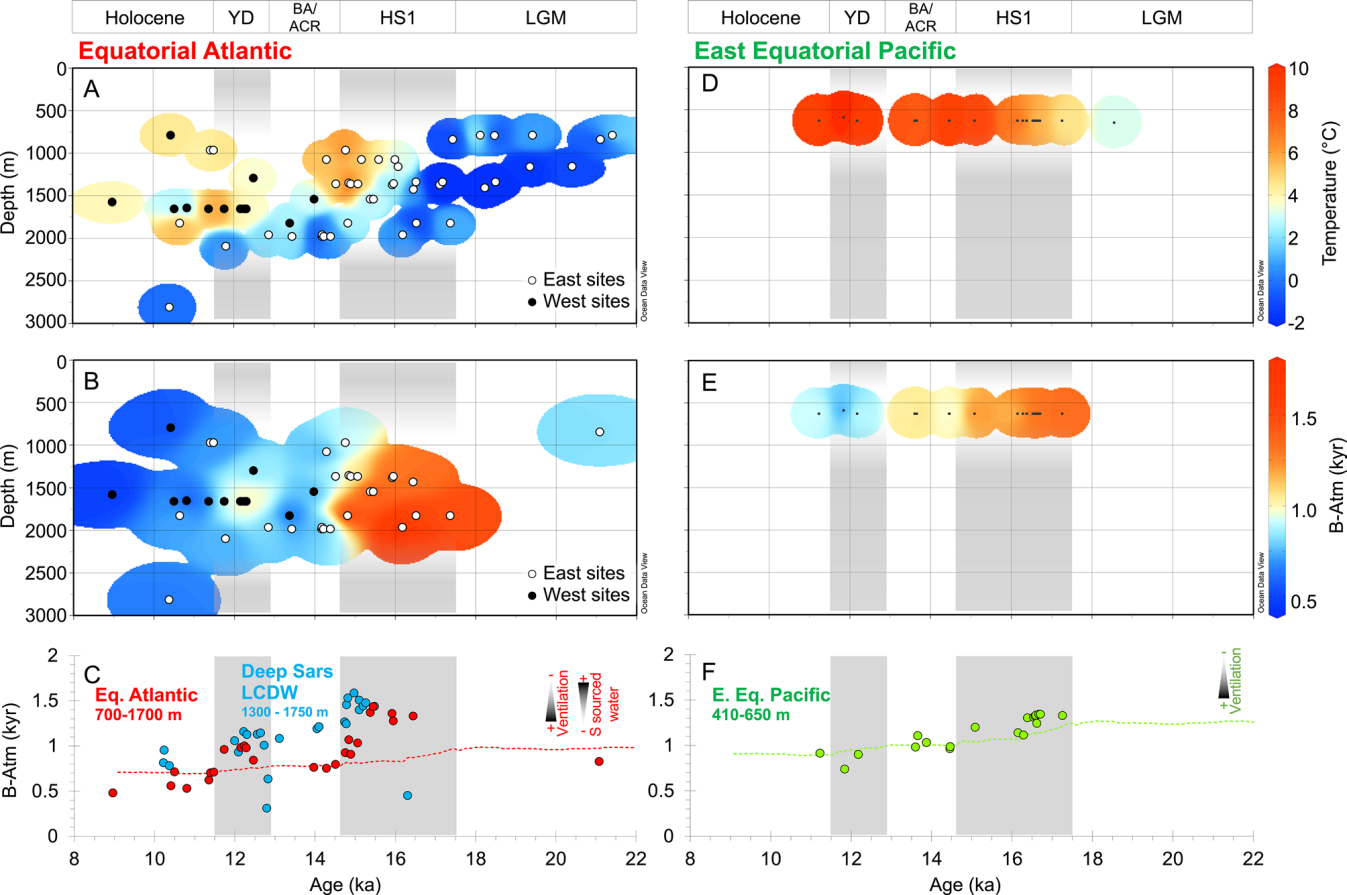
Hovmöller diagrams of coral temperature and radiocarbon content with water depth. (**A** and **D**) Coral temperature reconstruction for Equatorial Atlantic and Pacific (as in Fig. 4). Previous radiocarbon records from corals in this study from the Equatorial Atlantic (**B** and **C**)^36^, and East Equatorial Pacific (**E** and **F**)^40^. Equatorial Atlantic data are compared to deep Southern Ocean data (Sars Seamount^38^). Radiocarbon data are plotted as age difference between ^14^C sample age and contemporary atmosphere (“B-Atmosphere”). Dashed lines in (**C**) and (**F**) represent the modelled change in ^14^C content of seawater assuming atmospheric pCO_2_ is the only factor affecting ^14^C-reservoir age^40,43^.

We find the magnitude of Equatorial Atlantic warming during HS1 is large (~ + 6 °C increase)—similar to records from the nearby Demerara Rise based on the Li/Mg ratio of benthic foraminifera^22^ (Fig. 3D). Absolute temperature values reach ~ 5–8 °C by late HS1 (14.5 ka), warmer than equivalent waters in the Southern Ocean at this time. The depth of warm water incursion during HS1 also reaches down to ~ 1700 m, deeper than is typically associated with southern-sourced intermediate waters (Fig. 4). Similar “deep warming” has been recorded in temperature records from the Brazil Margin (warming at > 1200 m depth), where paired δ^13^C and Cd/Ca data suggest that warming also extended well below AAIW depths in the South Atlantic^16^. Radiocarbon data from the same Equatorial Atlantic corals show high B-Atm excursions (i.e. reduced radiocarbon content that deviates from the general increase in ocean ∆^14^C expected from more efficient air-sea carbon isotope exchange due to rising CO_2_^43^) towards values more similar to corals living in LCDW of the Southern Ocean during mid HS1 (Fig. 5C;^36,38^). However, by late HS1 these warmest waters in are younger (15–14.5 ka; ~ 1200 m depth; low B-Atm) than their Southern Ocean counterparts (compare Fig. 5A and B^36,38^). When taken together with evidence for (1) only modest deglacial warming of AAIW source waters and the Southern Ocean later into HS1 (Fig. 3G–I), (2) warmer absolute temperatures in intermediate depth waters in the Equatorial Atlantic than the Southern Ocean, (3) intense deepening of the upper ocean warmth (Fig. 4), and (4) slight cooling of Equatorial Atlantic waters into the Bølling–Allerød, these data suggest that the bulk of the deglacial warming of the Equatorial Atlantic is unlikely to be explained by advection of heat via southern-sourced intermediate waters and should instead be attributed to downward accumulation of heat in the absence of AMOC cooling at depth (“Warming 1”; Fig. 2).

In the Atlantic, the two hypothesised warming mechanisms are not mutually exclusive, and heat advected via southern-sourced intermediate waters (“Warming 2”; Fig. 2;^8,13^) would likely have amplified intermediate water warming at times during the deglaciation. Moreover, modelling suggests that warming of the Atlantic, down to depths of 1900 m, many have been further boosted by southward advection of North Atlantic mid-depth heat anomalies during this interval of diminished AMOC^8,16^. Indeed, spatial differences between downward and advected warming may account for differences in the magnitude of warming experienced at different Atlantic sites (Fig. 3D and E). Build-up of heat in the subsurface due to the removal of the cooling influence of NADW would also have played a role in surface water warming documented during HS1 and YD^44–48^. Regardless of the mechanism, warm waters in the ocean interior may have contributed to melting of glacial grounding lines—thus continuing ice sheet collapse—while its enhanced buoyancy as it reached depth would likely have preconditioned the Atlantic system for AMOC resumption at the end of HS1 (e.g.^20,36^).

The deglacial warming we document during HS1 at ~ 600 m water depth in the EEP is in contrast to distinct lack of warming recorded in benthic foraminiferal δ^18^O recovered from similar depths, just 180 km away, also on the Galápagos Platform (617 m; Core CDH 41^25^). Instead our coral temperature reconstructions closely track the timing of warming recorded in deeper EEP foraminiferal δ^18^O records (1023 m water depth; + 3 °C warming; Fig. 3C;^25^) and provide evidence that this deeper warming signal permeated the upper water column. Our EEP record is also synchronous with surface water warming reconstructed from foraminiferal Mg/Ca in this region (+ 2 to 3 °C warming^49–51^). Moreover, climate models suggest that such significant warming of EEP waters is indeed required to create the low pressure anomalies needed to explain southerly shifts in centres of precipitation towards the Andean regions of South America during HS1^52^.

Unlike in the Atlantic, poorly ventilated North Pacific Deep Water^53^ (Fig. 2) does not provide the necessary contrast in ^14^C in the EEP to identify the presence of poorly ventilated southern-sourced intermediate waters. Instead, B-Atm measurements in the same EEP corals used in this study are negatively correlated with reconstructed temperature (R^2^ = 0.9; Supplementary Fig. 2) as they track a generally smooth increase across the deglaciation towards more ventilated values (Fig. 5D–F^40^). This radiocarbon shift can be largely accounted for by an increase in the air-sea carbon isotope exchange efficiency under increasing *p*CO_2_^43^.

It is difficult to confirm or refute the presence of a strong Southern Ocean connection to the EEP via intermediate waters (“Warming 2”) during HS1 and YD using coral radiocarbon data alone, however other records suggest it played a role. Authigenic neodymium isotope records support advection of southern-sourced intermediate waters to the EEP during the last deglaciation. With the exception of the late Bølling–Allerød (13 ka), relatively low radiogenic neodymium isotope values recorded at thermocline depths (ODP Site 1240) suggest the presence of deglacial intermediate waters from the south in the EEP; particularly during HS1^11^. HS1 and YD are also associated with high diatom to coccolith ratios in this same sediment core, again suggesting that southern-sourced intermediate water—rich in nutrients (e.g. silicic acid)—reached the EEP at these times (Supplementary Fig. 1G;^54^). Such a connection has also been invoked to explain similarities between late deglaciation to Holocene subsurface temperature records in this region and southern high-latitude climate (Peru Margin benthic foraminifera Mg/Ca combined with δ^13^C;^55^). However, given the later onset and more limited warming we document in our Southern Ocean sites during HS1, additional warming mechanisms are potentially required to account for the full + 6 °C warming we observe in the EEP.

Both CCSM3^8^ and TRACE^16^ climate models support our interpretation that AMOC decline was the dominant driver of ocean warming in the Equatorial Atlantic. However, these same simulations predict that the effects of AMOC decline reached far beyond the Atlantic; also warming the South and East Equatorial Pacific by around + 2 °C, even down to 1000 m water depth^8,16^. The same dual warming mechanisms (“Warming 1” and “Warming 2”) may therefore help to explain the full extent of intermediate water warming we find in the EEP. Moreover, density gradients in the EEP likely reduced at the onset of the deglaciation, destratifying the water column, thus promoting vertical mixing and penetration of surface ocean warmth to depth^25^. Multiple foraminiferal geochemistry records from nearby sediment cores support this theory. For instance, U/Ca ratios and infaunal to epifaunal δ^13^C gradients^56^ and boron isotope records (ODP Site 1238)^51^ suggest that mid-depths became better oxygenated and this site once again became a region of deep ocean CO_2_ release at the onset of HS1.

## Conclusions

Our U-series-dated intermediate water coral temperature records from the East Equatorial Pacific, Equatorial Atlantic, and Southern Ocean provide new constraints on the commencement of the deglacial sub-surface warming. We find similar timing and magnitude of warming between our two tropical sites, particularly during HS1. These warming patterns happen synchronously with warming documented on Antarctica, suggesting that this high-latitude climate signal may have been partially communicated to the ocean interior via intermediate waters originating from the Southern Ocean. Despite these similarities, we find evidence for only modest deglacial warming of the Southern Ocean and a more abrupt and later warming of AAIW source waters at Burdwood Bank only mid-way through HS1. Intense deepening of upper ocean warmth in the Equatorial Atlantic during HS1 (and potentially the YD), below typical AAIW depths, further suggests that an additional warming mechanism is required. We suggest that downward accumulation of heat as a result of AMOC slowdown was also a dominant means of warming these tropical sites. Overall, our findings highlight the important role of the oceans in the communication of high-latitude climate anomalies and the redistribution of global heat through the ocean interior. This will have important implications for the ocean’s continued absorption of excess heat as a result of anthropogenic carbon emissions^57^.

## Materials and methods

Fossil scleractinian corals were collected from the Galápagos platform in the EEP (0° N, 90° E) by both dredging and remotely operated vehicle on cruises MV1007 and NA064 from water depths currently bathed by AAIW between 419 and 650 m (Fig. 2). Equatorial Atlantic corals (taxa *Caryophyllia, Enallopsammia, Desmophyllum*) were collected by remotely operated vehicle from a depth range of 749 to 2814 m during Cruise JC094 from Carter Seamount (9.2° N, 21.3° W), Knipovich Seamount (5.6° N, 26.9° W), Vema Fracture Zone (10.7° N, 44.6° W), Vayda Seamount (14.9° N, 48.2° W) and Gramberg Seamount (15.4° N, 51.1° W) (Fig. 2). Corals from between 700 and 1700 m water depth are classed as “intermediate” water while deeper samples 1800–2100 m water depth are classed as “deep” water (modern North Atlantic Deep Water). A single deeper-water sample from 2814 m (dated 10.4 ka) was also included in the “deep” classified samples.

Southern Ocean samples were obtained from Burdwood Bank (54.7° S, 62.2° W; taxa *Caryophyllia, Balanophyllia, Flabellum, Desmophyllum*) and Cape Horn (57.2° S, 67.1° W; taxa *Caryophyllia, Balanophyllia, Flabellum*) in the Subantarctic Zone and the Sars and Interim Seamounts in the Polar Front Zone (59.7° S, 68.8° W and 60.6° S, 66.0° W; taxa *Caryophyllia, Desmophyllum*) on cruises NBP0805 and NBP1103 in the Drake Passage (Fig. 2). These proximal Sars and Interim sites are grouped as simply “Sars” for discussion. The shallowest coral samples come from depths of 334 m on Burdwood Bank however the majority are from 700 to 1520 m, at water depths corresponding to modern AAIW. Corals recovered from the depth of 1012 m from Cape Horn and further south from Sars Seamount at depths of 695–1200 m are currently bathed in UCDW. Deeper samples at the Sars Seamount site sit within LCDW (1300–1750 m).

We use published U-series dates for all samples^36–41^. Reported age uncertainties are typically ± 1% (2 SD).

**Figure 6:**
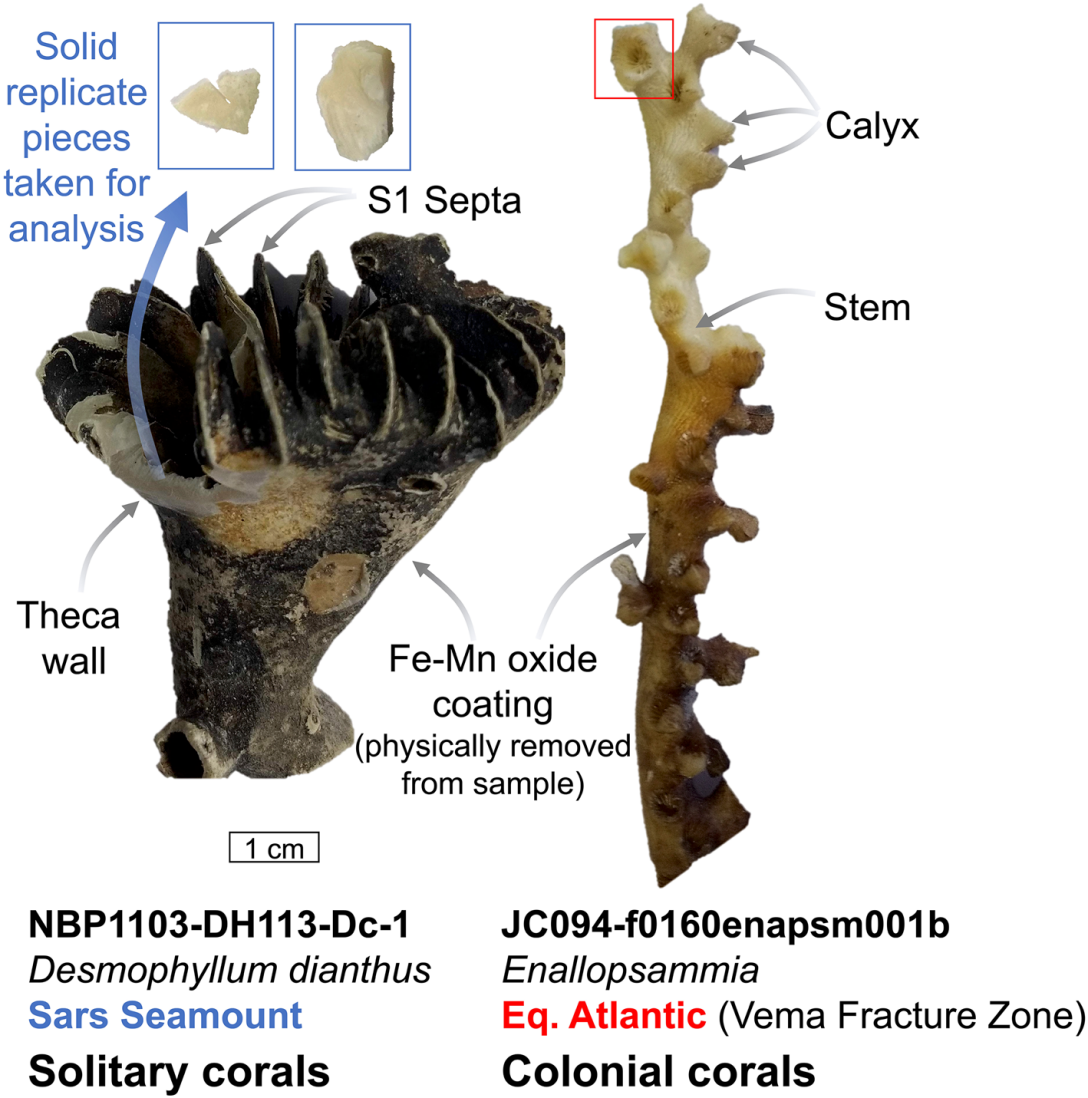
Sampling strategy of solitary and colonial corals in this study. For individual replicate samples whole “S1” septa and associated theca wall were cut from solitary corals (e.g. *Desmophyllum*) whereas whole calyxes were taken from colonial corals (e.g. *Enallopsammia*) to be representative of bulk coral chemistry.

Whole “S1” septa^58^ and attached theca were taken from cup corals while whole calyxes were taken from branching specimens using a rotary cutting tool (Fig. 6). This tool was further used to remove surficial oxide coatings and any chalky altered carbonate. Microstructures within cold-water corals are known to exhibit contrasting skeletal chemistry^59^, therefore where sufficient sample material allowed, multiple sub-samples were measured to minimize microstructural bias (typically duplicates; open symbols in results Fig. 1). Coral fragments were finely crushed using a pestle and mortar before a 5 mg aliquot of the powder was taken. A warm 1% H_2_O_2_ (buffered in NH_4_OH) oxidative clean and weak acid polish (0.0005 M HNO_3_) was performed on powders^60,61^. Samples were dissolved in distilled 0.5 M HNO_3_.

Analyses were carried out at the Universities of Bristol or St Andrews. An aliquot of the dissolved sample was analyzed by ICP-MS using well-characterised, matrix-matched, synthetic standard solutions to yield Li/Mg ratios. Repeat analysis of NIST RM 8301 (Coral) (n = 19) yielded analytical precision of <  ± 1.5%. Small analytical offsets between the labs are quantified and corrected using NIST RM 8301 (Coral) results to bring values into line with interlaboratory comparison results^62^.

Coral Li/Mg was converted to temperature using a calibration applicable to all aragonitic corals (Li/Mg = 5.42 exp(− 0.050 × T(°C));^35^. By using this technique there are no “vital effect” offsets to adjust for between different coral taxa (Supplementary Fig. 3). The quoted uncertainty on this calibration based on prediction intervals is ± 1.7 °C (1σ). This uncertainty is significantly reduced however at extremely low temperatures close to the freezing point of seawater (~ − 2 °C). Corals could not survive in frozen seawater, therefore, where proxy estimated temperature falls below this minimum (e.g. some Last Glacial Maximum samples with Li/Mg ≥ 6 mmol/mol; Fig. 1), a value of − 2 °C is reported instead (Fig. 3).

### Supplementary Information

Supplementary Information 1.

Supplementary Information 2.

## Data Availability

All data generated during this study are included in this published article and its supplementary information files. Data are also available from the Pangaea database (10.1594/PANGAEA.964080).

## References

[1] Kindler P (2014). Temperature reconstruction from 10 to 120 kyr b2k from the NGRIP ice core. Clim. Past.

[2] Parrenin F (2013). Synchronous change of atmospheric CO_2_ and antarctic temperature during the last deglacial warming. Science.

[3] Buizert C (2015). The WAIS Divide deep ice core WD2014 chronology—part 1: Methane synchronization (68–31 ka BP) and the gas age–ice age difference. Clim. Past.

[4] Broecker WS (1998). Paleocean circulation during the last deglaciation: A bipolar seesaw?. Paleoceanography.

[5] Böhm E (2015). Strong and deep Atlantic meridional overturning circulation during the last glacial cycle. Nature.

[6] McManus JF, Francois R, Gherardi JM, Keigwin LD, Brown-Leger S (2004). Collapse and rapid resumption of Atlantic meridional circulation linked to deglacial climate changes. Nature.

[7] Ng HC (2018). Coherent deglacial changes in western Atlantic Ocean circulation. Nat. Commun..

[8] Pedro JB (2018). Beyond the bipolar seesaw: Toward a process understanding of interhemispheric coupling. Quat. Sci. Rev..

[9] Barker S (2009). Interhemispheric Atlantic seesaw response during the last deglaciation. Nature.

[10] Rae JWB (2018). CO_2_ storage and release in the deep Southern Ocean on millennial to centennial timescales. Nature.

[11] Pena LD (2013). Rapid changes in meridional advection of Southern Ocean intermediate waters to the tropical Pacific during the last 30kyr. Earth Planet. Sci. Lett..

[12] Romahn S, Mackensen A, Groeneveld J, Pätzold J (2014). Deglacial intermediate water reorganization: New evidence from the Indian Ocean. Clim. Past.

[13] Marcott SA (2011). Ice-shelf collapse from subsurface warming as a trigger for Heinrich events. Proc. Natl. Acad. Sci..

[14] Galbraith ED, Merlis TM, Palter JB (2016). Destabilization of glacial climate by the radiative impact of Atlantic Meridional Overturning Circulation disruptions. Geophys. Res. Lett..

[15] Hain MP, Sigman DM, Haug GH (2014). Distinct roles of the Southern Ocean and North Atlantic in the deglacial atmospheric radiocarbon decline. Earth Planet. Sci. Lett..

[16] Umling NE (2019). Atlantic circulation and ice sheet influences on upper South Atlantic temperatures during the last deglaciation. Paleoceanogr. Paleoclimatol..

[17] Poggemann DW (2018). Deglacial heat uptake by the southern ocean and rapid northward redistribution via antarctic intermediate water. Paleoceanogr. Paleoclimatol..

[18] Weldeab S, Friedrich T, Timmermann A, Schneider RR (2016). Strong middepth warming and weak radiocarbon imprints in the equatorial Atlantic during Heinrich 1 and Younger Dryas. Paleoceanography.

[19] Roberts J (2016). Evolution of South Atlantic density and chemical stratification across the last deglaciation. Proc. Natl. Acad. Sci..

[20] Thiagarajan N, Subhas AV, Southon JR, Eiler JM, Adkins JF (2014). Abrupt pre-Bølling–Allerød warming and circulation changes in the deep ocean. Nature.

[21] Hines SKV, Eiler JM, Southon JR, Adkins JF (2019). Dynamic intermediate waters across the late glacial revealed by paired radiocarbon and clumped isotope temperature records. Paleoceanogr. Paleoclimatol..

[22] Oppo DW (2023). Deglacial temperature and carbonate saturation state variability in the tropical atlantic at antarctic intermediate water depths. Paleoceanogr. Paleoclimatol..

[23] Valley SG, Lynch-Stieglitz J, Marchitto TM (2019). Intermediate water circulation changes in the Florida Straits from a 35 ka record of Mg/Li-derived temperature and Cd/Ca-derived seawater cadmium. Earth Planet. Sci. Lett..

[24] Stott L, Southon J, Timmermann A, Koutavas A (2009). Radiocarbon age anomaly at intermediate water depth in the Pacific Ocean during the last deglaciation. Paleoceanography.

[25] Bova SC (2015). Links between eastern equatorial Pacific stratification and atmospheric CO2 rise during the last deglaciation. Paleoceanography.

[26] Pahnke K, Goldstein SL, Hemming SR (2008). Abrupt changes in Antarctic Intermediate Water circulation over the past 25,000 years. Nat. Geosci..

[27] Huang K-F, Oppo DW, Curry WB (2014). Decreased influence of Antarctic intermediate water in the tropical Atlantic during North Atlantic cold events. Earth Planet. Sci. Lett..

[28] Xie RC, Marcantonio F, Schmidt MW (2012). Deglacial variability of Antarctic Intermediate Water penetration into the North Atlantic from authigenic neodymium isotope ratios. Paleoceanography.

[29] Gu S (2017). Coherent response of Antarctic intermediate water and Atlantic meridional overturning circulation during the last deglaciation: Reconciling contrasting neodymium isotope reconstructions from the Tropical Atlantic. Paleoceanography.

[30] Poggemann D-W (2017). Rapid deglacial injection of nutrients into the tropical Atlantic via Antarctic Intermediate Water. Earth Planet. Sci. Lett..

[31] Rickaby REM, Elderfield H (2005). Evidence from the high-latitude North Atlantic for variations in Antarctic Intermediate water flow during the last deglaciation. Geochem. Geophys. Geosyst..

[32] Valley S, Lynch-Stieglitz J, Marchitto TM (2017). Timing of deglacial AMOC variability from a high-resolution seawater cadmium reconstruction. Paleoceanography.

[33] Came RE, Oppo DW, Curry WB, Lynch-Stieglitz J (2008). Deglacial variability in the surface return flow of the Atlantic meridional overturning circulation. Paleoceanography.

[34] Yu J (2019). More efficient North Atlantic carbon pump during the last glacial maximum. Nat. Commun..

[35] Stewart JA (2020). Refining trace metal temperature proxies in cold-water scleractinian and stylasterid corals. Earth Planet. Sci. Lett..

[36] Chen T (2015). Synchronous centennial abrupt events in the ocean and atmosphere during the last deglaciation. Science.

[37] Margolin AR (2014). Temporal and spatial distributions of cold-water corals in the Drake Passage: Insights from the last 35,000 years. Deep Sea Res. Part II: Top. Stud. Oceanogr..

[38] Li T (2020). Rapid shifts in circulation and biogeochemistry of the Southern Ocean during deglacial carbon cycle events. Sci. Adv..

[39] Burke A, Robinson LF (2012). The Southern Ocean’s role in carbon exchange during the last deglaciation. Science.

[40] Chen T (2020). Persistently well-ventilated intermediate-depth ocean through the last deglaciation. Nat. Geosci..

[41] Stewart JA (2021). Productivity and dissolved oxygen controls on the southern ocean deep-sea benthos during the antarctic cold reversal. Paleoceanogr. Paleoclimatol..

[42] Oppo DW, Lehman SJ (1993). Mid-depth circulation of the subpolar North Atlantic during the last glacial maximum. Science.

[43] Hain MP, Sigman DM, Haug GH (2011). Shortcomings of the isolated abyssal reservoir model for deglacial radiocarbon changes in the mid-depth Indo-Pacific Ocean. Geophys. Res. Lett..

[44] Weldeab S, Schneider RR, Kölling M (2006). Deglacial sea surface temperature and salinity increase in the western tropical Atlantic in synchrony with high latitude climate instabilities. Earth Planet. Sci. Lett..

[45] Schmidt MW (2012). Impact of abrupt deglacial climate change on tropical Atlantic subsurface temperatures. Proc. Natl. Acad. Sci..

[46] Naafs BDA, Hefter J, Grützner J, Stein R (2013). Warming of surface waters in the mid-latitude North Atlantic during Heinrich events. Paleoceanography.

[47] Meier KJF (2021). Role of the tropical Atlantic for the interhemispheric heat transport during the last deglaciation. Paleoceanogr. Paleoclimatol..

[48] Osman MB (2021). Globally resolved surface temperatures since the Last Glacial Maximum. Nature.

[49] Lea DW (2006). Paleoclimate history of Galápagos surface waters over the last 135,000 yr. Quat. Sci. Rev..

[50] Pena LD, Cacho I, Ferretti P, El Hall MA (2008). Niño-Southern Oscillation–like variability during glacial terminations and interlatitudinal teleconnections. Paleoceanography.

[51] Martínez-Botí MA (2015). Boron isotope evidence for oceanic carbon dioxide leakage during the last deglaciation. Nature.

[52] Zhang Y (2016). Equatorial Pacific forcing of western Amazonian precipitation during Heinrich Stadial 1. Sci. Rep..

[53] Olsen A (2016). The global ocean data analysis project version 2 (GLODAPv2)—an internally consistent data product for the world ocean. Earth Syst. Sci. Data.

[54] Calvo E, Pelejero C, Pena LD, Cacho I, Logan GA (2011). Eastern Equatorial Pacific productivity and related-CO_2_ changes since the last glacial period. Proc. Natl. Acad. Sci..

[55] Kalansky J, Rosenthal Y, Herbert T, Bova S, Altabet M (2015). Southern Ocean contributions to the Eastern Equatorial Pacific heat content during the Holocene. Earth Planet. Sci. Lett..

[56] Umling NE, Thunell RC (2018). Mid-depth respired carbon storage and oxygenation of the eastern equatorial Pacific over the last 25,000 years. Quat. Sci. Rev..

[57] Rhein, M. *et al.* In *Climate Change 2013: The Physical Science Basis. Contribution of Working Group I to the Fifth Assessment Report of the Intergovernmental Panel on Climate Change* (eds T.F. Stocker *et al.*) Ch. 3, 255–316 (Cambridge University Press, 2013).

[58] Cairns SD (1994). Scleractinia of the Temperate North Pacific.

[59] Blamart D (2007). Correlation of boron isotopic composition with ultrastructure in the deep-sea coral *Lophelia pertusa*: Implications for biomineralization and paleo-pH. Geochem. Geophys. Geosyst..

[60] Boyle EA (1981). Cadmium, zinc, copper, and barium in foraminifera tests. Earth Planet. Sci. Lett..

[61] Rae JWB, Foster GL, Schmidt DN, Elliott T (2011). Boron isotopes and B/Ca in benthic foraminifera: Proxies for the deep ocean carbonate system. Earth Planet. Sci. Lett..

[62] Stewart JA (2020). NIST RM 8301 boron isotopes in marine carbonate (simulated coral and foraminifera solutions): Inter-laboratory δ^11^B and trace element ratio value assignment. Geostandard. Geoanal. Res..

[63] Buizert C (2015). Precise interpolar phasing of abrupt climate change during the last ice age. Nature.

[64] Bereiter B (2014). Revision of the EPICA Dome C CO_2_ record from 800 to 600 kyr before present. Geophys. Res. Lett..

[65] Schlitzer, R. *Ocean Data View, Version 4.6.5. *http://odv.awi.de (2021).

