# Supplementary material for: Arctic and Antarctic forcing of ocean interior warming during the last deglaciation
Source: Sci Rep. 2023 Dec 16;13:22410. doi: 10.1038/s41598-023-49435-0 (PMC10725493; doi:10.1038/s41598-023-49435-0)
Supplement: Supplementary file 2 — Supplementary Information 2. [file 41598_2023_49435_MOESM2_ESM.docx]

**Arctic and Antarctic forcing of ocean interior warming during the last deglaciation**

**Joseph A. Stewart ^1^, Laura F. Robinson ^1^, James W. B. Rae ^2^, Andrea Burke ^2^, Tianyu Chen ^1,3^, Tao Li ^1,3^, Maria Luiza de Carvalho Ferreira ^1^,** **Daniel J. Fornari ^4^**

^1^ School of Earth Sci. Univ. of Bristol, Queens Road, Bristol, BS8 1RJ, UK

^2^ Earth & Environmental Sci., Univ. of St Andrews, Bute Building, KY16 9TS

^3^ School of Earth Sciences and Engineering, Nanjing University, Nanjing 210023, China

^4^ Woods Hole Oceanographic Institution, MA, USA

Correspondence [joseph.stewart@bristol.ac.uk](mailto:joseph.stewart@bristol.ac.uk)

Supplementary information


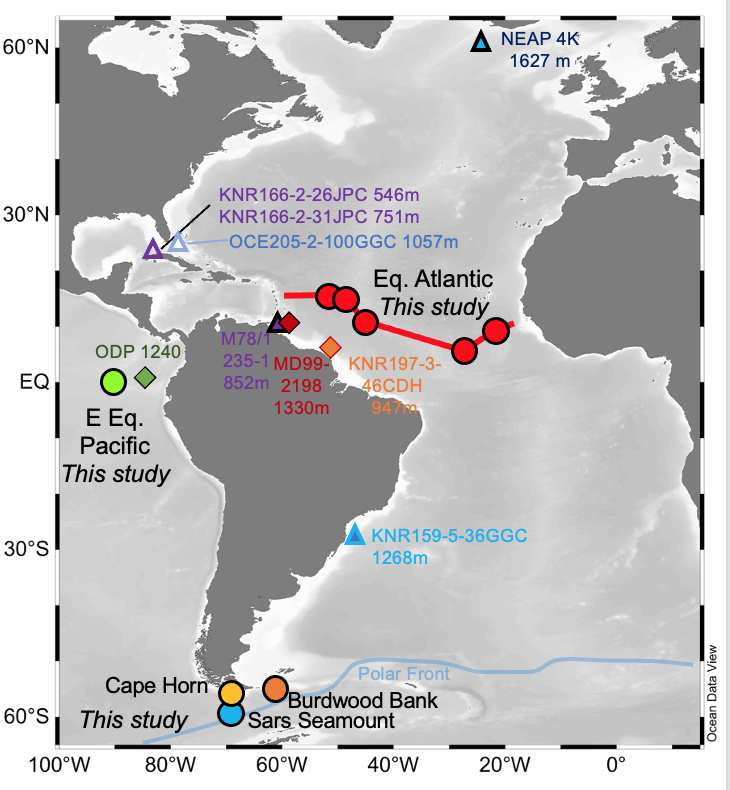


**Supplementary Figure 1:** **Comparison to circulation proxy data** (**A**) Antarctic ice core temperature change and (**B**) atmospheric CO_2_ records ^1-3^. (**C**) Compiled δ^13^C of atmospheric CO_2_ from ice cores ^4^ and δ^13^C of Atlantic intermediate water dissolved inorganic carbon from benthic foraminifera ^5^. White arrows highlight the divergence of these δ^13^C records at 17 ka. (**D, E & F**) Previous radiocarbon data for corals in this study ^6-9^: (**D**) EEP, (**E**) Equatorial Atlantic compared to deep Southern Ocean samples (deep Sars), (**F**) Burdwood Bank and Cape Horn (orange arrow highlights the reorganisation of ^14^C in the Southern Ocean recorded at the same time as abrupt warming on Burdwood Bank). Radiocarbon data are plotted as age difference between ^14^C sample age and contemporary atmosphere (“B-Atmosphere”). Dashed lines represent the modelled change in ^14^C content of seawater assuming atmospheric pCO_2_ is the only factor affecting ^14^C-reservoir age ^8,10^. (**G**) East Equatorial Pacific (ODP Site 1240) diatom to coccolith ratio measure of surface water primary production ^11^. (**H)** Seawater neodymium isotope (water mass proxy) reconstructions from Fe-Mn coatings from KNR197-3-46CDH, 947 m water depth ^12^ and MD99-2198, 1330 m water depth ^13^. (**I** & **J**) Cadmium concentration of seawater from Cd/Ca of benthic foraminifera (nutrient proxy) from cores KNR166-2-26JPC (546 m; ^14^); KNR166-2-31JPC (751 m ^15^); KNR159-5-36GGC (1268 m; ^16^); M78/1 235-1 (852 m water depth; ^17^); NEAP 4K (1627 m; ^18^); OCE205-2-100GGC (1057 m; ^15^). (**K**) Coral Li/Mg intermediate water temperature reconstruction (this study) for low latitude East Equatorial Pacific and Equatorial Atlantic sites. Map drawn using Ocean Data View ^19^.

**Supplementary Figure 2:** **Deglacial tropical intermediate water temperature (this study) compared to (A) Antarctic temperature**^3^ **and (B) coral radiocarbon content**^6,8^**.** Coral temperature data in this study are matched to the nearest Antarctic temperature value by age. Radiocarbon data again shown as “B-Atmosphere”. **(A)** Tropical intermediate waters from both the Equatorial Atlantic (red) and East Equatorial Pacific (green) both correlate strongly with Antarctic temperatures during the last deglaciation. **(B)** Equatorial Atlantic coral temperature shows poor correlation with B-Atmosphere, whereas East Equatorial Pacific coral temperatures are tightly negatively correlated with B-Atmosphere. Data labels on East Equatorial Pacific in (B) represent coral ages (ka) and are colour coded to represent early (black), middle (green), and late (yellow) deglaciation. This radiocarbon shift in the Pacific can be largely accounted for by an increase in the air-sea carbon isotope exchange efficiency under increasing *p*CO_2_ ^10^, whereas intermittent AAIW influence (depleted in ^14^C; high B-Atm) may have disrupted this relationship in the Atlantic.


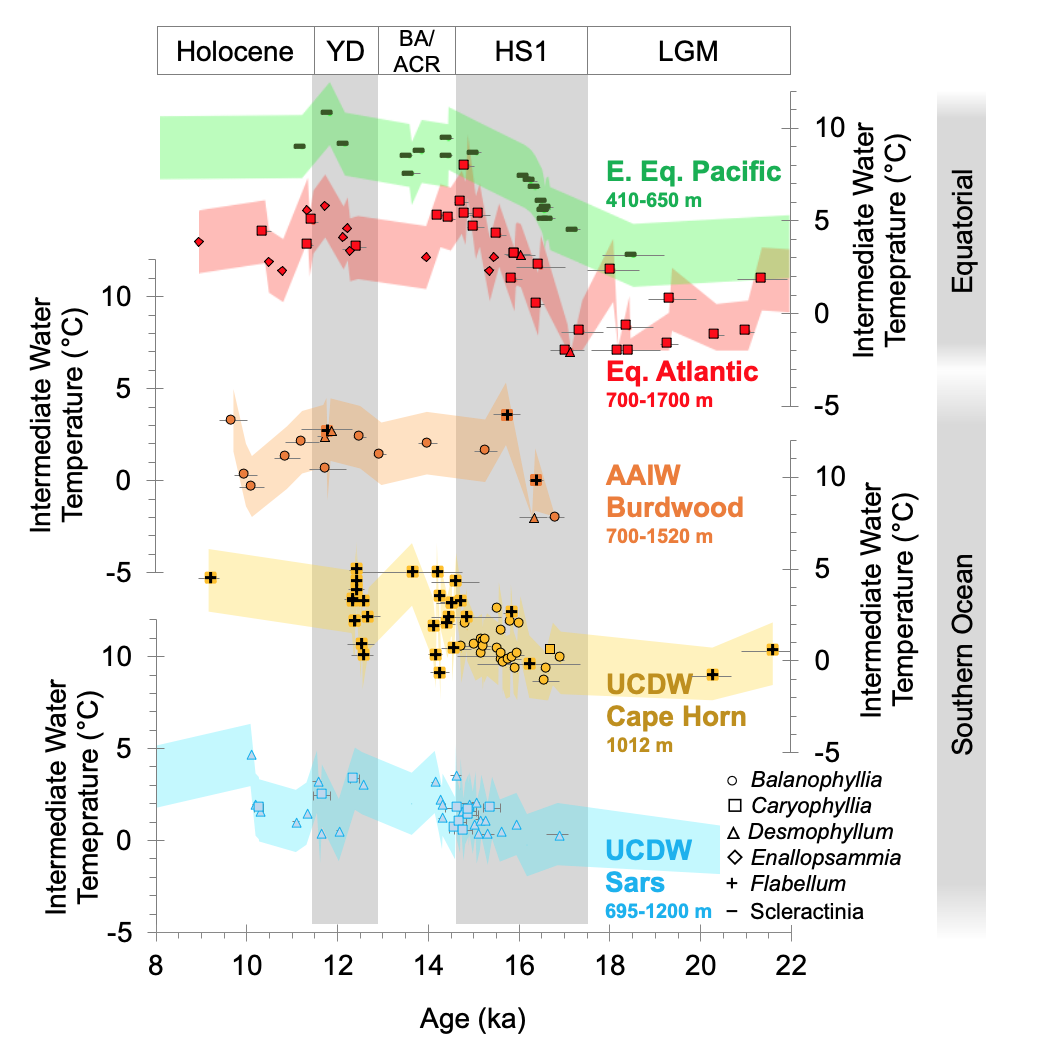


**Supplementary Figure 3:** **Coral temperature records by coral taxon.** Li/Mg temperature proxy data in this study. Symbols denote coral genus of scleractinian coral (where available). The Li/Mg proxy calibration^20^ used in this study applies to all aragonitic (i.e. scleractinian) coral taxa and species offsets do not need to be corrected for. For this reason, filtering data by coral taxa has little impact on the findings of this study.

*Implications of warming for benthic δ^13^C records*

Records of seawater stable carbon isotopes have long been used as a water mass tracer in the Atlantic for detecting the presence of southern-sourced waters rich in respired carbon (^12^C-enriched) ^21,22^. Shifts towards low δ^13^C have been documented at intermediate water depths in the tropical (e.g. ^23^) and far north Atlantic (e.g. 60 °N; ^18^) during HS1 and YD. While these records potentially indicate the presence of AAIW at these sites, seawater δ^13^C can also be influenced by changes in local carbon cycling and temperature ^5^. Indeed, our finding that warming of the Equatorial Atlantic commenced at 17 ka has important implications (Supplementary Figure 1K). The δ^13^C values of dissolved inorganic carbon compiled across multiple Atlantic intermediate water sites decreased by 0.5 ‰ relative to atmospheric CO_2_ δ^13^C at 17 ka ^4,5^ (Supplementary Figure 1C). The equilibrium partitioning of stable carbon isotopes between the atmosphere and seawater is temperature dependent, therefore this decrease could be interpreted as either widespread release of respired (^12^C-enriched) carbon early in the deglaciation (e.g. ^24^) or a warming of sub-surface Atlantic waters of ~5°C ^5^. Our tropical Atlantic temperature records imply that large magnitude sub-surface warming commencing at 17 ka may have been sufficient to account for the full divergence between benthic δ^13^C and the atmosphere in this region.

References

1 Bereiter, B. *et al.* Revision of the EPICA Dome C CO_2_ record from 800 to 600 kyr before present. *Geophysical Research Letters* **42**, 542-549, (2014).

2 Buizert, C. *et al.* Precise interpolar phasing of abrupt climate change during the last ice age. *Nature* **520**, 661-665, (2015).

3 Parrenin, F. *et al.* Synchronous Change of Atmospheric CO_2_ and Antarctic Temperature During the Last Deglacial Warming. *Science* **339**, 1060-1063, (2013).

4 Schmitt, J. *et al.* Carbon Isotope Constraints on the Deglacial CO_2_ Rise from Ice Cores. *Science* **336**, 711, (2012).

5 Lynch-Stieglitz, J., Valley, S. G. & Schmidt, M. W. Temperature-dependent ocean–atmosphere equilibration of carbon isotopes in surface and intermediate waters over the deglaciation. *Earth and Planetary Science Letters* **506**, 466-475, (2019).

6 Chen, T. *et al.* Synchronous centennial abrupt events in the ocean and atmosphere during the last deglaciation. *Science* **349**, 1537-1541, (2015).

7 Li, T. *et al.* Rapid shifts in circulation and biogeochemistry of the Southern Ocean during deglacial carbon cycle events. *Science Advances*, eabb3807, (2020).

8 Chen, T. *et al.* Persistently well-ventilated intermediate-depth ocean through the last deglaciation. *Nature Geoscience* **13**, 733-738, (2020).

9 Burke, A. & Robinson, L. F. The Southern Ocean’s Role in Carbon Exchange During the Last Deglaciation. *Science* **335**, 557-561, (2012).

10 Hain, M. P., Sigman, D. M. & Haug, G. H. Shortcomings of the isolated abyssal reservoir model for deglacial radiocarbon changes in the mid-depth Indo-Pacific Ocean. *Geophys. Res. Lett.* **38**, L04604, (2011).

11 Calvo, E., Pelejero, C., Pena, L. D., Cacho, I. & Logan, G. A. Eastern Equatorial Pacific productivity and related-CO_2_ changes since the last glacial period. *Proceedings of the National Academy of Sciences* **108**, 5537, (2011).

12 Huang, K.-F., Oppo, D. W. & Curry, W. B. Decreased influence of Antarctic intermediate water in the tropical Atlantic during North Atlantic cold events. *Earth and Planetary Science Letters* **389**, 200-208, (2014).

13 Pahnke, K., Goldstein, S. L. & Hemming, S. R. Abrupt changes in Antarctic Intermediate Water circulation over the past 25,000 years. *Nature Geoscience* **1**, 870, (2008).

14 Valley, S., Lynch-Stieglitz, J. & Marchitto, T. M. Timing of Deglacial AMOC Variability From a High-Resolution Seawater Cadmium Reconstruction. *Paleoceanography* **32**, 1195-1203, (2017).

15 Came, R. E., Oppo, D. W., Curry, W. B. & Lynch-Stieglitz, J. Deglacial variability in the surface return flow of the Atlantic meridional overturning circulation. *Paleoceanography* **23**, (2008).

16 Came, R. E., Oppo, D. W. & Curry, W. B. Atlantic Ocean circulation during the Younger Dryas: Insights from a new Cd/Ca record from the western subtropical South Atlantic. *Paleoceanography* **18**, (2003).

17 Poggemann, D.-W. *et al.* Rapid deglacial injection of nutrients into the tropical Atlantic via Antarctic Intermediate Water. *Earth and Planetary Science Letters* **463**, 118-126, (2017).

18 Rickaby, R. E. M. & Elderfield, H. Evidence from the high‐latitude North Atlantic for variations in Antarctic Intermediate water flow during the last deglaciation. *Geochemistry, Geophysics, Geosystems* **6**, (2005).

19 Schlitzer, R. *Ocean Data View, Version 4.6.5* [*http://odv.awi.de*](http://odv.awi.de), 2021).

20 Stewart, J. A. *et al.* Refining trace metal temperature proxies in cold-water scleractinian and stylasterid corals. *Earth and Planetary Science Letters* **545**, 116412, (2020).

21 Oppo, D. W., Curry, W. B. & McManus, J. F. What do benthic δ13C and δ18O data tell us about Atlantic circulation during Heinrich Stadial 1? *Paleoceanography* **30**, 353-368, (2015).

22 Oppo, D. W. & Fairbanks, R. G. Variability in the deep and intermediate water circulation of the Atlantic Ocean during the past 25,000 years: Northern Hemisphere modulation of the Southern Ocean. *Earth and Planetary Science Letters* **86**, 1-15, (1987).

23 Lynch-Stieglitz, J. *et al.* Muted change in Atlantic overturning circulation over some glacial-aged Heinrich events. *Nature Geoscience* **7**, 144, (2014).

24 Romahn, S., Mackensen, A., Groeneveld, J. & Pätzold, J. Deglacial intermediate water reorganization: new evidence from the Indian Ocean. *Clim. Past* **10**, 293-303, (2014).
